# Cryogenically preserved RBCs support gametocytogenesis of *Plasmodium falciparum* in vitro and gametogenesis in mosquitoes

**DOI:** 10.1186/s12936-018-2612-y

**Published:** 2018-12-06

**Authors:** Ashutosh K. Pathak, Justine C. Shiau, Matthew B. Thomas, Courtney C. Murdock

**Affiliations:** 1 Department of Infectious Diseases, College of Veterinary Medicine, University of Georgia, Athens, GA 30602 USA; 2 Center for Infectious Disease Dynamics and the Department of Entomology, Pennsylvania State University, State College, PA 16803 USA; 3 Odum School of Ecology, University of Georgia, Athens, GA 30602 USA; 4 Center for Ecology of Infectious Diseases, University of Georgia, Athens, GA 30602 USA; 5 Center for Tropical Emerging Global Diseases, University of Georgia, Athens, GA 30602 USA; 6 Center for Vaccines and Immunology, University of Georgia, Athens, GA 30602 USA; 7 Riverbasin Center, University of Georgia, Athens, GA 30602 USA

**Keywords:** *Anopheles stephensi*, Cryogenic preservation, Gametogenesis, Malaria, *Plasmodium falciparum*, RBCs, SMFAs, Transmission

## Abstract

**Background:**

The malaria Eradication Research Agenda (malERA) has identified human-to-mosquito transmission of *Plasmodium falciparum* as a major target for eradication. The cornerstone for identifying and evaluating transmission in the laboratory is standard membrane feeding assays (SMFAs) where mature gametocytes of *P. falciparum* generated in vitro are offered to mosquitoes as part of a blood-meal. However, propagation of “infectious” gametocytes requires 10–12 days with considerable physico-chemical demands imposed on host RBCs and thus, “fresh” RBCs that are ≤ 1-week old post-collection are generally recommended. However, in addition to the costs, physico-chemical characteristics unique to RBC donors may confound reproducibility and interpretation of SMFAs. Cryogenic storage of RBCs (“cryo-preserved RBCs”) is accepted by European and US FDAs as an alternative to refrigeration (4 °C) for preserving RBC “quality” and while cryo-preserved RBCs have been used for in vitro cultures of other *Plasmodia* and the asexual stages of *P. falciparum*, none of the studies required RBCs to support parasite development for > 4 days.

**Results:**

Using the standard laboratory strain, *P. falciparum* NF54, 11 SMFAs were performed with RBCs from four separate donors to demonstrate that RBCs cryo-preserved in the gaseous phase of liquid nitrogen (− 196 °C) supported gametocytogenesis in vitro and subsequent gametogenesis in *Anopheles stephensi* mosquitoes. Overall levels of sporogony in the mosquito, as measured by oocyst and sporozoite prevalence, as well as oocyst burden, from each of the four donors thawed after varying intervals of cryopreservation (1, 4, 8, and 12 weeks) were comparable to using ≤ 1-week old refrigerated RBCs. Lastly, the potential for cryo-preserved RBCs to serve as a suitable alternative substrate is demonstrated for a Cambodian isolate of *P. falciparum* across two independent SMFAs.

**Conclusions:**

Basic guidelines are presented for integrating cryo-preserved RBCs into an existing laboratory/insectary framework for *P. falciparum* SMFAs with significant potential for reducing running costs while achieving greater reliability. Lastly, scenarios are discussed where cryo-preserved RBCs may be especially useful in enhancing the understanding and/or providing novel insights into the patterns and processes underlying human-to-mosquito transmission.

**Electronic supplementary material:**

The online version of this article (10.1186/s12936-018-2612-y) contains supplementary material, which is available to authorized users.

## Background

Incidence of malaria due to *Plasmodium falciparum* has seen a steady decline in the last 15–20 years, enabled primarily by the synergy between drug-combination therapies for case/disease management and vector control programmes [1, 2]. Since transmission to the mosquito is thought to be a severe bottleneck in the parasite’s life history, considerable effort has been channeled towards identifying parasite and vector traits influencing transmission to the mosquito with the aim of designing and testing small molecule inhibitors and vaccines [3–6]. The experimental model for evaluating basic parasite life history or the efficacy of various interventions typically involve standard-membrane feeding assays (SMFAs), where mature male and female gametocytes of *P. falciparum* cultured in vitro are supplemented with naïve RBCs and offered as part of a blood-meal to female *Anopheles* spp. mosquitoes. These assays are then followed by the collection of various measures of parasite fitness in the mosquito vector [7–11]. However, in the 40 years since Trager and Jensen’s first description of the methodology, in vitro cultures of transmission-competent parasite stages (the definitive measure of parasite fitness) have proven to be an arduous and labour-intensive venture with the precise trigger(s) for induction of gametocytogenesis remaining largely elusive [12–15]. Currently available methodology is a synthesis of several tools and techniques that have been elegantly summarized in two recent publications [13, 15].

Relative to other etiological agents of human malaria, *P. falciparum* is unique in its life-history in blood wherein the asexual replication period lasts 2 days during which a variable, but relatively small fraction of the resulting parasite progeny is pre-destined to invade an RBC and enter an irreversible path of sexual differentiation lasting an additional 10–12 days before maturation into male and female gametocytes capable of infecting a mosquito. Most references outlining methods for growing sexual stages in RBCs in vitro emphasize the need for maintaining the sexual stages in fresh RBCs (i.e., storage under refrigeration for no more than 1-week from the day of collection), as the RBC must be able to support gametocytogenesis both physically and energetically during the 10 to 12-day differentiation period [13, 15–18]. However, ensuring a regular supply of commercially sourced RBCs can quickly prove to be prohibitively expensive. Additionally, variation introduced by differences in storage time of RBCs and across blood donors can potentially carry significant consequences for the reproducibility of downstream experiments and across laboratories for validating vaccine and drug efficacy for instance [9, 19–21].

Despite > 100 years of experience in bio-banking, identifying the true “shelf-life” of refrigerated RBCs is controversial even in case of human blood transfusions [22]. While it may be possible to restore some aspects of RBC metabolism upon rejuvenation of packed RBCs with the addition of fresh nutrient-containing media, most of the storage-associated changes are simply irreversible [23–25]. As an alternative, cryo-preservation of RBCs in glycerol-based cryo-protectants (storage at − 65 °C or below) has been suggested using procedures approved by both the United States Food and Drug Administration and the European Council [26, 27].

While cryo-preserved RBCs had been shown to support the growth of other aetiological agents of human malaria, such as *Plasmodium vivax*, a recent study demonstrated that growth rates of the asexual blood stages of *P. falciparum* in cryogenically-preserved RBCs (− 196 °C in the gaseous phase of liquid nitrogen) was almost identical to freshly collected blood in vitro [28, 29]. However, none of these studies required the RBCs to sustain parasite growth for more than 4 days. According to current FDA guidelines, cryo-preserved RBCs thawed under sterile conditions can retain physicochemical properties for up to (at least) 14 days following the thaw date [26]. Considering how this timeframe encompasses the duration required for gametocytogenesis of *P. falciparum*, cryo-preserved RBCs should, hypothetically, also provide a suitable substrate for the culture of sexual stages of *P. falciparum*. Moreover, as successful parasite establishment and infection in the mosquito vector is the definitive measure of parasite fitness, the suitability of cryo-preserved RBCs to support gametocytogenesis, gametogenesis, and sporogony in *Anopheles stephensi* was assessed. Additionally, whether infectiousness was retained following extended periods of cryo-preservation of RBCs was addressed with RBCs preserved from independent donors (see Additional file 1 for a schematic of study objectives, Additional file 2 for donor-specific testing regime and schedule/design). Lastly, although these objectives were addressed primarily with the standard laboratory strain (NF54) of *P. falciparum*, the potential for cryo-preserved RBCs to serve as a substrate for supporting growth and differentiation of infectious stages of other strains of the parasite was verified with a Cambodian isolate of *P. falciparum*, CB132 [30].

## Methods

### Study design

The overall study design is described below, and in the supplement accompanying this manuscript (Additional file 1). Cryo-preservation was performed 3–4 days after collection as suggested elsewhere [27, 31]. To control for the variability inherent to SMFAs [9] and thus account for any technical/biological variation arising from unknown factors independent of RBC storage method and duration, the study was originally designed with the goal of meeting two important criteria. First, gametocyte flasks for comparing each treatment were initiated from the same asexual seed culture since parasite fitness can vary over generations, which in turn can introduce variation in induction of gametocytogenesis. Second, infectious cultures resulting from the treatments were offered to mosquitoes that were sorted from the same starting population and age post-emergence to minimize any variation in sporogony caused by inter-generational differences across mosquito cohorts. Therefore, each experimental block was composed of the following steps: (1) the *same asexual seed culture* was used to initiate gametocytogenesis, (2) rates of gametocytaemia monitored over 14–16 days in individual flasks (i.e., 1 flask/treatment) with refrigerated (4 °C) and/or cryo-preserved RBCs at various storage durations, and (3) sexually mature gametocytes from each flask/treatment offered to *mosquitoes from the same cohort* to assess the efficiency of gametogenesis and sporogony.

Although the experimental design was initially centered on replicating the outcomes across 4 unique donor RBC populations while fulfilling the criteria outlined above, a variety of unforeseen logistical constraints (e.g. in vitro contamination) prevented the accomplishment of these goals (Additional file 2). As such, the experimental design was “partially nested” wherein not all 4 donors were represented equally across all treatments of storage method or duration and to account for this, all statistical analyses were performed using generalized linear mixed-effects models (GLMMs) that are particularly well-suited for analysing such datasets, as suggested previously [32–34] (see Additional file 2 for schematic showing donor-specific testing regime within experimental blocks).

### Chemicals and consumables

All reagents and consumables described herein were purchased from Fisher Scientific (Hampton, NH) unless noted otherwise.

### Preparation of media components

Parasite culture media was prepared and stored as described previously [15], with minor modifications. Briefly, incomplete media was prepared by dissolving pre-made RPMI-1640 powdered media in distilled water before adding 2% sodium bicarbonate (w/v) and 0.005% hypoxanthine (w/v, Sigma-Aldrich, St. Louis, MO). The incomplete media was then filter-sterilized with 0.2 µm filters under vacuum before moving to storage at − 20 °C in aliquot sizes of 450 or 900 mls. Complete media was prepared just prior to use by adding 50 or 100 mls of 10% A+ non-heat inactivated human serum (Valley Biomedical, Winchester, VA) to 450 or 900 mls to thawed, incomplete media respectively. Media was dispensed into 45 ml aliquots in 50 ml conical-bottom tubes and the air–liquid interface sparged for 5–10 s with a micro-aerophilic gas mixture of 5% CO_2_, 5% O_2_ and 90% N_2_ (referred to herein as “tri-gas mixture”, Airgas LLC, Kennesaw, GA) prior to storage for 1 week. Since “quality” of serum is critical for culturing transmission competent parasites, serum samples were tested as described previously [15]. Serum from 14 individuals was pooled into pairs and tested for their ability to support gametocytogenesis as well as ex-flagellation of male gametocytes in case of *P. falciparum* isolate NF54 (unpublished observations). The same pool of serum donors was used to culture *P. falciparum* NF54 for the duration of this study.

### Cryo-preservation and thawing of RBCs

Cryo-preservation of RBCs was performed 3–4 days after collection as suggested elsewhere [27, 31]. Additionally, it should be noted that due to practical constraints, the comparison between ≤ 1-week old refrigerated and cryo-preserved RBCs were performed after the latter had been stored for 2–3 days.

Procedures for freezing and thawing were adapted from Goheen et al. [28] and Sputtek [31] with minor modifications. Two-fold concentrated stocks of cryo-protectant were prepared in deionized water by warming a solution of 28% glycerol (v/v), 3% sorbitol (v/v from a 1 M stock) and 0.65% (w/v) sodium chloride (NaCl) prior to sterilization with a 0.2 µm filter and storage at room temperature. Whole blood (~ 500 ml units, Valley Biomedical, Winchester, VA or Interstate Blood Bank, Memphis, TN) was dispensed in 45 ml aliquots and either stored refrigerated at 4 °C until use (see next section) or allowed to warm to room temperature prior to cryo-preservation. To ensure reproducible recovery of viable RBCs following cryo-preservation, it is critical for the cryo-protectant and RBCs to be at the same temperature (e.g., room temperature), prior to use [31].

Aliquots of whole blood, no more than 3–4 days post-collection, were centrifuged at 1800×*g* for 10 min at room temperature at a low brake setting and plasma and white blood cell layers aspirated under vacuum before an equal volume of cryo-protectant solution (~ 20–25 mls) was added to the packed RBC pellet to achieve a final glycerol concentration of 14% (v/v) and a haematocrit of ~ 50%. The packed RBCs were then equilibrated with the cryo-protectant for 15–20 min with gentle intermittent mixing. Glycerolized RBCs were then dispensed in 2 ml aliquots and snap-frozen by immersing in liquid nitrogen for 2–3 min before transferring to the vapour phase for long-term storage. Cryo-preserved RBCs were thawed just before use by incubating cryogenic vials in a dry bath for 5 min at 37 °C before transferring the contents into a 15 or 50 ml conical bottom tube prior to de-glycerolization. Unless stated otherwise, all solutions for de-glycerolization were prepared in deionized water and sterilized with 0.2 µm filters before storage at room temperature. De-glycerolization was initiated with the addition of successive gradients of sodium chloride concentrations starting with 0.4 mls of 12% NaCl (w/v in distilled water, 0.2 volumes for every ml of RBC and cryoprotectant mixture) added dropwise under gentle agitation (~ 1 min). The mixture was then incubated at room temperature for 5 min with gentle intermittent mixing before dropwise addition of 10 mls (5 volumes) of sterile 1.6% NaCl (w/v in distilled water). The de-glycerolized RBC suspension was centrifuged at 1000×*g* for 3 min at 20–23 °C and low brakes and 20mls (10 volumes) of a salt-dextrose solution (0.9% NaCl + 0.2% dextrose, w/v in distilled water) added dropwise to the packed RBC pellet under gentle agitation. If haemolysis was still observed in the supernatant, the packed RBCs were washed once more with the same volume of salt-dextrose solution. Finally, the thawed RBCs were resuspended in complete media before use. Recoveries of ~ 0.8–0.9 mls of packed RBCs should be considered routine.

### Preparation of refrigerated RBCs

On the day of use, but ≤ 1-week post-collection, a 45 ml aliquot of whole blood was retrieved from the refrigerator and allowed to warm to room temperature before centrifugation as described in the preceding section. The plasma and white blood cell layers were aspirated before the addition of an equal volume of incomplete media (~ 20–25 mls), also at room temperature. RBCs were gently re-suspended in incomplete media before centrifugation for 3 min at 1000×*g* and low brake setting. Packed RBCs were washed two more times before re-suspension in an equal volume of complete media to achieve a final haematocrit of ~ 50%.

### Cultures of asexual stage *P. falciparum*

*Plasmodium falciparum* isolate NF54 was obtained from BEI Resources, NIAID, NIH (‘*Plasmodium falciparum*, Strain NF54 (Patient Line E), product number MRA-1000, contributed by Megan G. Dowler’). Asexual feeder cultures were routinely initiated at ring state parasitaemia of 0.1–1% using cryo-preserved RBCs pooled from 2 to 4 donors as substrate and final haematocrit of 5% in 5 or 15 mls of complete media for T25 or T75 flasks, respectively. The same volume of media was replaced every 24 h and then sparged gently at the air–liquid interface with the tri-gas mixture for 15–20 s. Parasitaemia was monitored every 1–2 days by transferring 50–100 µl of parasite culture to a 0.6 ml tube, centrifuged at 1800×*g* for 1 min and supernatants aspirated until final culture volume was ~ 15–20 µl. The RBC pellet was re-suspended with repeated pipetting before preparing smears with 2 µl of the concentrate on glass slides. Slides were dried on a slide-warmer for 20–30 s and then fixed by submerging in 100% Methanol for 10 s prior to Giemsa staining. Concentrated Giemsa (Sigma-Aldrich, St. Louis, MO) was filtered in aliquot sizes of 30–35 mls with a Whatman no. 1 filter paper and stored at room temperature. Just before use, the filtered stain was diluted to a ratio of 1:20 (v/v) in phosphate-buffered saline (pH 7.2) and fixed slides immersed in the stain in glass Coplin-jars for 10–15 min at room temperature. Stained sides were washed under running tap-water for 5–10 s with the smeared side facing downwards before being air-dried for microscopy. Smears were mounted directly in immersion oil (Cargille Labs, Cedar Grove, NJ) and examined at 1000× magnification with a Leica DM2500 upright microscope (Leica Microsystems, Buffalo Grove, IL). Parasitaemia was estimated from ~ 1000 RBCs and staged into early trophozoites (referred to herein as rings), late trophozoites and schizonts following guidelines described previously [13, 35]. Ring-stage parasitaemia expressed as a proportion of RBCs counted was used to prepare flasks for routine sub-culture and/or gametocytogenesis [15].

### Cultures of the sexual stages (gametocytes) of *Plasmodium falciparum*

Flasks destined for gametocytogenesis were only initiated when ring-stage parasitaemia in the asexual seed flasks above showed exponential increase in growth rates (> 6- to 8-fold) relative to the previous sampling point. Flasks for gametocytogenesis were prepared essentially as described above with 1 flask representing each treatment (Additional file 5a). Briefly, freshly thawed or refrigerated RBCs resuspended in pre-warmed (37 °C) complete media at 5% haematocrit (e.g., 1 mls naïve RBCs in 20 mls complete media) were seeded with asexual cultures to achieve a final ring-stage density of 0.6–1% in the gametocyte flasks and returned to an incubator maintained at 37 °C. Media in flasks was replaced every 24 h with 15 mls of fresh pre-warmed (37 °C) complete media and sparged with tri-gas mixture for 10–30 s. All manipulations outside the incubator were performed by placing the flask on a slide-warming platform maintained at 38 °C (MedSupply Partners, Atlanta, GA). Gametocyte flasks were maintained for 14–16 days and parasitaemia monitored with Giemsa staining at 1-, 4-, 8-, 12- and 14-days post-seeding as suggested previously [15]. Asexual stages were staged as described above and gametocytes were further classified into stages II, III, IV and V male or V female gametocytes using guidelines established previously [13, 35] (Additional file 3a, b). Stage I gametocytes were excluded from the counts as they are virtually indistinguishable morphologically from late trophozoites, especially in asynchronous cultures [36]. Finally, maturation status of the culture was assessed from 12 days post-seeding by quantifying ex-flagellation of male stage V gametocytes in a Neubauer haemocytometer chamber as suggested previously [15]. Briefly, after adding fresh media, 10 µl of culture was pipetted directly into the chambers of a haemocytometer and incubated at 21–24 °C for 15–20 min before quantification (Additional file 3c). Imaging was performed at 400× magnification on a Leica DM2500 equipped with differential interference contrast (DIC) optics. A flask was deemed infectious once ex-flagellation was observed and offered to mosquitoes within 24–48 h.

*Plasmodium falciparum* Cambodian isolate CB132 (kind gift of Prof. Dennis E Kyle, University of Georgia, Athens, GA, USA) [30] was cultured under similar conditions to NF54 with the exception that flasks destined for gametocytogenesis were seeded at a density of 0.5%.

### Mosquito husbandry

*Anopheles stephensi* mosquitoes were maintained in a level 2 Arthropod Containment Laboratory at the University of Georgia, which were initiated from eggs kindly provided by the Walter Reed Army Institute of Research ca. 2015 is a wild-type strain referred to as Strain “Indian” [10, 37]. Colonies were housed in a dedicated walk-in environmental chamber [chamber (Percival Scientific, Perry, IA)] at 27 °C ± 0.5 °C, 80% ± 5% relative humidity, and under a 12 h light: 12 h dark photo-period schedule. Adult mosquitoes were maintained on 5% dextrose (w/v) and 0.05% para-Aminobenzoic acid (w/v) and provided whole human blood offered in glass-jacketed feeders (Chemglass Life Sciences, Vineland, NJ) through parafilm membrane maintained at 37 °C to support egg production. Husbandry procedures were established according to guidelines as suggested elsewhere [38] with minor modifications. Briefly, eggs were rinsed twice with 1% house-hold bleach (v/v, final concentration of 0.06% sodium hypochlorite) before surface-sterilization for 1 min in the same solution at room temperature. Bleached eggs were washed with 4–5 changes of deionized water and transferred to clear plastic trays (34.6 cm L × 21.0 cm W × 12.4 cm H) containing 500 ml of deionized water and 2 medium pellets of Hikari Cichlid Gold fish food (HikariUSA, Hayward, CA) and allowed to hatch for 48 h. Hatched L1 larvae were dispensed into clear plastic trays (34.6 cm L × 21.0 cm W × 12.4 cm H) at a density of 300 larvae/1000 ml water and provided the same diet until pupation. The feeding regime consisted of 2 medium pellets provided on the day of dispensing (day 0) followed by the provision of a further 2, 4, 4 and 4 medium pellets on days 4, 7, 8 and 9 respectively. This regime allows > 85% larval survival and > 90% pupation of the surviving larvae within 11 days with a sex ratio of 1:1 adult males and females (unpublished observations).

### Mosquito infections

All steps outlined below were performed with pre-warmed (38 °C) equipment, including tubes, pipettes, pipette tips and centrifuge rotor buckets. Cultures deemed infectious were concentrated by centrifugation at 1800×*g* for 1–2 min at room temperature in pre-weighed 15 or 50 ml conical bottom centrifuge tubes. Supernatants were aspirated under vacuum and the weight/volume of the concentrate estimated by weighing the tube and subtracting the value of the pre-weighed empty tube. 3–6 volumes of a freshly washed and pre-warmed mixture of RBCs resuspended in freshly thawed serum (30% haematocrit) was added to the infected RBC pellet to achieve a final haematocrit of 45–50% and mature gametocytaemia of ~ 0.1%. The volumes of naïve RBCs and serum mixture to be added was informed by the densities recorded in the gametocyte flasks on the day of infection. The mixture of naïve and parasitized RBCs was then fed immediately to 60–80 (3–7 day old) female *An. stephensi* mosquitoes for 15–20 min. To facilitate high blood feeding rates, mosquitoes were previously starved for 16–24 h in dedicated environmental chambers programmed to fluctuate a total of 9 °C around a daily mean of 24 °C with 80% ± 5% relative humidity and a 12 h light:dark photo-period schedule (Percival Scientific, Perry, IA) as described previously [10]. After the blood-feeds, feeding status was qualitatively ascertained and engorged mosquitoes selected for by extending the starvation period for a further 24–48 h to eliminate non-blood-fed mosquitoes [39].

To quantify parasite densities in the infectious feed, Giemsa-stained smears were prepared from a 2 µl aliquot of the infectious blood meal offered to mosquitoes, as described above. Prevalence and abundance of parasites in the midguts of mosquitoes was assessed at 9–13 days post-infection by dissecting midguts and counting oocysts at 400× magnification with a Leica DM2500 under DIC optics (Additional file 3d). Sporozoite prevalence was assessed at 17–21 days post-infection by dissecting salivary glands into 5 µl of PBS. Glands were ruptured by overlaying a 22 mm^2^ coverslip and presence/absence of sporozoites was recorded for each mosquito at either 100× or 400× magnification with the same microscope (Additional file 3e).

### Statistical modelling and data analyses

All data analyses were performed in RStudio (version 1.1.423) [40] running the statistical software package R (version 3.5) [41]. Choice of GLMM family and corresponding link function were based on five dependent variables modelled—(1) rates of mature gametocytaemia in vitro, (2) oocyst prevalence (proportion of mosquitoes with oocysts on the midgut), (3) oocyst abundance (total number of oocysts per midgut regardless of infection status), (4) oocyst intensity (number of oocysts per midgut from infected midguts only), and (5) sporozoite prevalence (proportion of mosquitoes with sporozoites in the salivary glands). For all the analyses, storage method (refrigerated vs. cryo-preserved RBCs) and duration (4, 8, and 12 weeks post cryo-preservation) were specified as independent variables, with storage method and duration classified as a categorical and continuous predictor, respectively. Storage duration was centred and scaled over the grand mean. For analysing temporal patterns of gametocytogenesis in vitro, GLMMs with a binomial distribution (“logit” link) were performed with the total count of RBCs infected with male and female stage V gametocytes (i.e., total mature gametocytes) expressed as a proportion of the sum of RBCs (infected and uninfected) counted from each sample as the dependent variable. Oocyst and sporozoite prevalence in the midguts and salivary glands of mosquitoes, respectively, were modelled as the probability of mosquitoes being infected and infectious as dependent variables in GLMMs specified with a binomial distribution (“logit” link function). For analysing oocyst abundance and intensity, GLMMs with negative binomial distributions (“log” link) were utilized. All statistical analyses were performed using the package “lme4” unless stated otherwise [42]. For the in vitro analysis, the intercepts of the relationships between the rates of mature gametocytaemia and the predictor variables was allowed to vary between RBC donors as well as over sampling period (days post-seeding) between experimental blocks and flasks nested within each block. The same random effect structure was specified for analysing parasite fitness in the mosquitoes except infections/SMFAs were nested within blocks *in lieu* of flasks (Additional files 1 and 2). Tabulation of model outputs, along with tests for overdispersion were performed with the “sjstats” package. Where applicable, post hoc comparisons of storage method and duration were performed using Tukey contrasts of the estimated marginal means derived from the models above, as suggested in the “emmeans” package [43].

Finally, cross-validation of the models were performed by (1) testing fit after truncating the number of mosquitoes sampled across each infection and (2) removing experimental block(s) and/or flasks/infections within each block. Where applicable, random effects were checked for normal distribution patterns and the final output tables of statistical modelling prepared with the “sjPlot” package and “sjstats” packages [44, 45]. All figures were prepared using the “ggplot2” package [46].

## Results

### Gametocytogenesis of *Plasmodium falciparum* in vitro

**Fig. 1:**
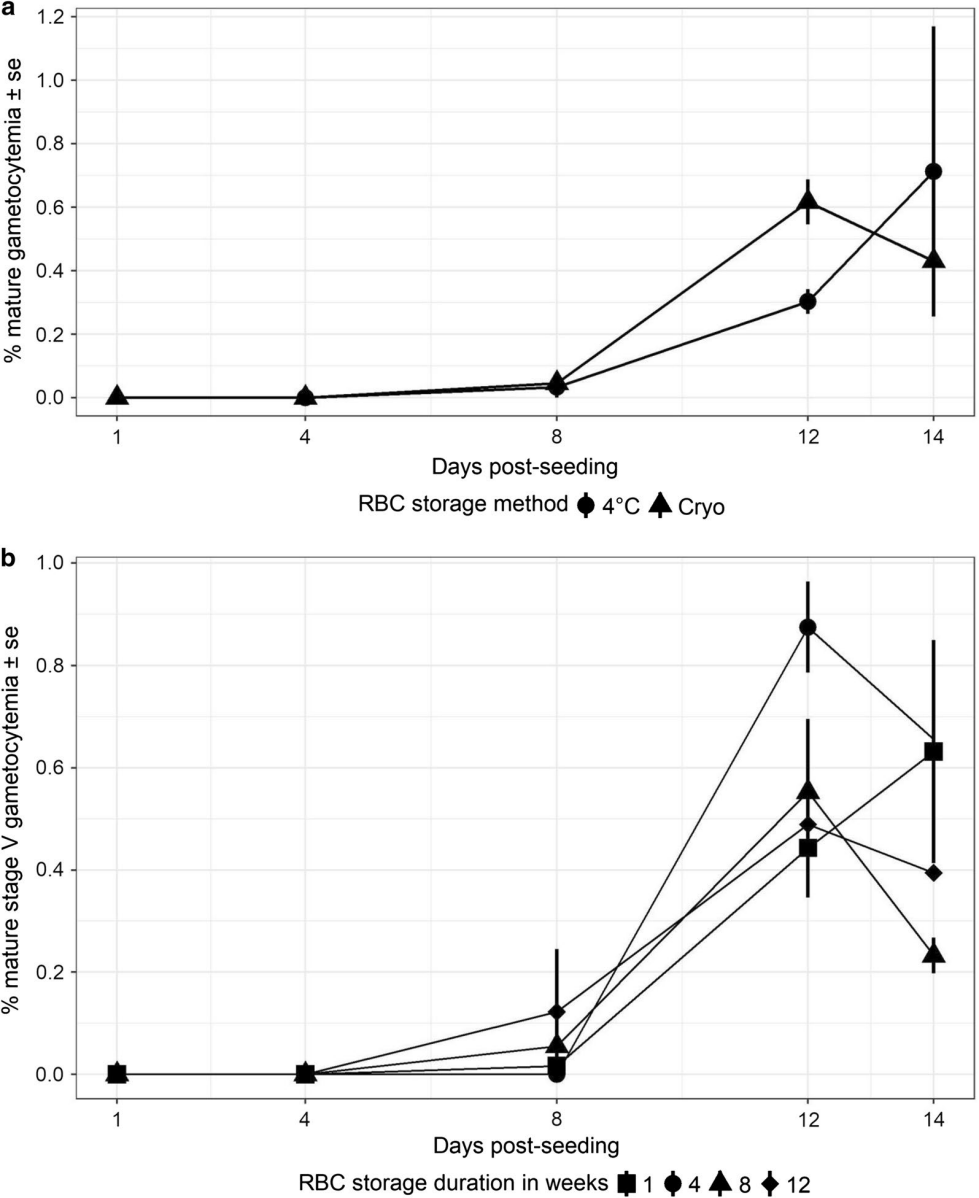
**a** Overall rates of mature gametocytaemia of *Plasmodium falciparum* NF54 cultured in vitro in refrigerated (closed circles) or cryo-preserved (closed triangles) RBCs and **b** rates of gametocytaemia in RBCs cryo-preserved within 3–4 days of collection from each donor and thawed after 1 (closed squares), 4 (closed circles), 8 (closed triangles) and 12 weeks (closed diamonds) of storage. Data represents mean ± standard errors (se). See Additional file 3a for detailed trends separated by flask, RBC donor as well as experimental block

The influence of storage method (cryo-preserved RBCs vs. the reference standard of refrigerated RBCs ≤ 1-week post-collection) was assessed for the rates of gametocytogenesis in vitro (Fig. 1, Additional file 3a and b for images of mature gametocytes and Additional file 4 for developmental rates of preceding immature stages II–IV). Mature gametocytaemia in vitro generally increased over time with detectable levels achieved by 12 days post-seeding (Fig. 1). Overall trends were independent of storage method (Fig. 1a and Table 1, Odds ratios (OR) = 1.31, standard error (se) = 0.41, *p *= 0.4) or duration (Fig. 1b and Table 1, OR = 1.01, se = 0.04, *p *= 0.72) with most of the random variation explained by differences between experimental block (Table 1 and Additional file 5a) and negligible contribution by RBC donor (Fig. 1b, Table 1 and Additional file 5a).

**Table 1:** Outputs of the statistical models

Dependent variable →	Gametocytemia in vitro (male + female stage V)			Oocyst prevalence in mosquito midguts			Sporozoite prevalence in salivary glands			Oocyst abundance (infected and uninfected midguts)			Oocyst intensity (infected midguts only)		
Summary statistics →	OR	Std. error	p-value	OR	Std. error	p-value	OR	Std. error	p-value	IRR	Std. error	p-value	IRR	Std. error	p-value
Fixed effects															
(Intercept)	0.00	0.00	< 0.001	1.09	0.52	0.86	0.82	0.50	0.75	1.96	0.85	0.43	5.19	0.48	< 0.001
4 °C RBCs vs Cryo-RBCs	1.31	0.41	0.40	1.05	0.38	0.89	1.35	0.84	0.63	1.20	0.71	0.80	1.12	0.36	0.75
Duration of storage	1.01	0.04	0.72	0.89	0.06	0.07	0.93	0.08	0.36	0.86	0.10	0.13	0.95	0.07	0.48
Experimental design															
Total flasks/mosquito infections (SMFAs), nested within experimental block	11			11			10^a^			11			11		
Total number of experimental blocks	5			5			5			5			5		
Total no. of RBC donors tested	4			4			4			4			4		
Total number of observations	51			283			235			283			157		
Random effects															
Group structure and variance for random effects															
Flasks/SMFAs nested within experimental block	0.00			0.00			0.26			0.43			0.10		
Experimental block	0.16			0.35			0.00			0.77			0.40		
RBC donor	0.00			0.17			0.02			0.46			0.21		
Overdispersion tests															
Dispersion ratio	0.98			1.23			1.00			0.75			1.07		
p-value	0.50			0.29			0.40			1.00			0.27		
Notes															
GLMM family (link)	Binomial (logit)			Binomial (logit)			Binomial (logit)			Negative binomial (log)			Negative binomial (log)		

*IRR* Incidence rate ratios, *NA* not applicable, *OR* odds ratios

^a^No of infections is lower as sporozoite prevalence was not available from 1 infection

### Prevalence of *Plasmodium falciparum* in midguts of *Anopheles stephensi* mosquitoes

**Fig. 2:**
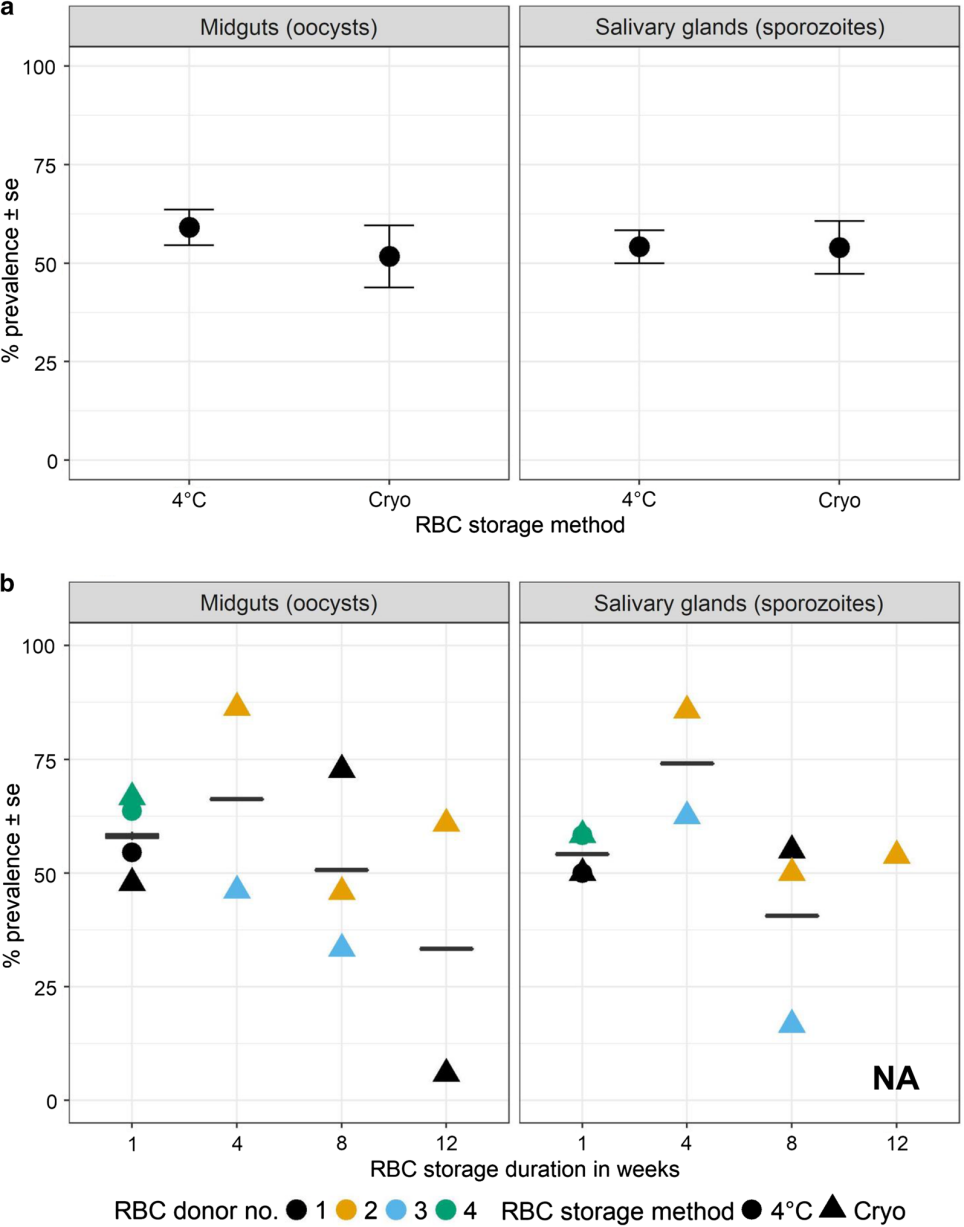
**a** Overall prevalence of oocysts and sporozoites in midguts (left panel) and salivary glands (right panel) of *Anopheles stephensi* mosquitoes infected with mature gametocytes of *Plasmodium falciparum* NF54 from flasks depicted in Fig. 1. Data represents mean ± se. **b** Prevalence of oocysts and sporozoites from parasites cultured in RBCs collected from four independent donors (indicated by colours) stored for 1-week as refrigerated (“4 °C”, circles) or cryo-preserved (“Cryo”, triangles). NA = not available because sporozoite prevalence for donor 1 was zero due to low corresponding oocyst prevalence at the preceding midgut stages (6%, closed black triangle in b, left panel). Horizontal bars represent group means. See Additional file 3b for prevalence separated by RBC donor and experimental block

To determine if the sexually mature gametocytes generated in vitro are infectious, sporogony in *An. stephensi* mosquitoes was assessed, starting with oocyst prevalence in the midguts (infected/total midguts) at time points corresponding to peak stages of infection (Additional file 3d). The mean prevalence of mosquitoes infected with *P. falciparum* oocysts (NF54) was 53 ± 6.4% (mean ± se, n = 11 SMFAs, mean mosquito midguts sampled/SMFA = 26 (range = 17–44), total number of mosquito midguts sampled = 283) with 59 ± 4.6% (n = 2) and 52 ± 7.9% (n = 9) in mosquitoes infected with parasites cultured in refrigerated and cryo-preserved RBCs, respectively (Fig. 2a, left panel). While prevalence was independent of storage method (Fig. 2a, left panel and Table 1, OR = 1.05, se = 0.38, *p *= 0.89), the ability of cryo-preserved RBCs to culture infectious gametocytes showed a marginally insignificant decline with duration of storage (Fig. 2b, left panel and Table 1, OR = 0.89, se = 0.06, *p *= 0.07) with much of the unexplained variation represented by differences across experimental blocks (Table 1 and Additional file 5b), although some could also be attributed to RBC donor characteristics (Fig. 2b, left panel, Table 1 and Additional file 5b).

### Prevalence of *Plasmodium falciparum* in the salivary glands of mosquitoes

To verify sporozoite egress from the oocyst and completion of the sporogonic cycle, salivary glands from the same cohort of mosquitoes were dissected and scored for the presence or absence of sporozoites (Additional file 3e). The mean (± se) prevalence of mosquitoes carrying sporozoites of *P. falciparum* NF54 in the salivary glands was 54 ± 5.3% (n = 10 SMFAs, mean salivary glands sampled/SMFA = 24 (range = 20–30), total number of salivary glands sampled = 235) with 54 ± 4.2% (n = 2) and 54% ± 6.8% (n = 8) in mosquitoes infected with parasites cultured in refrigerated and cryo-preserved RBC, respectively (Fig. 2a, right panel). Overall trends in sporozoite prevalence were independent of storage method (Fig. 2a, right panel and Table 1, OR = 1.35, se = 0.84, p = 0.63) or duration (Fig. 2b, right panel and Table 1, OR = 0.93, se = 0.08, p = 0.36). Unlike the midguts however, most of the random variation was caused by differences among infections with negligible contributions from experimental block or RBC donor (Fig. 2b, right panel and Table 1).

### Oocyst abundance and intensity in the midguts of mosquitoes

**Fig. 3:**
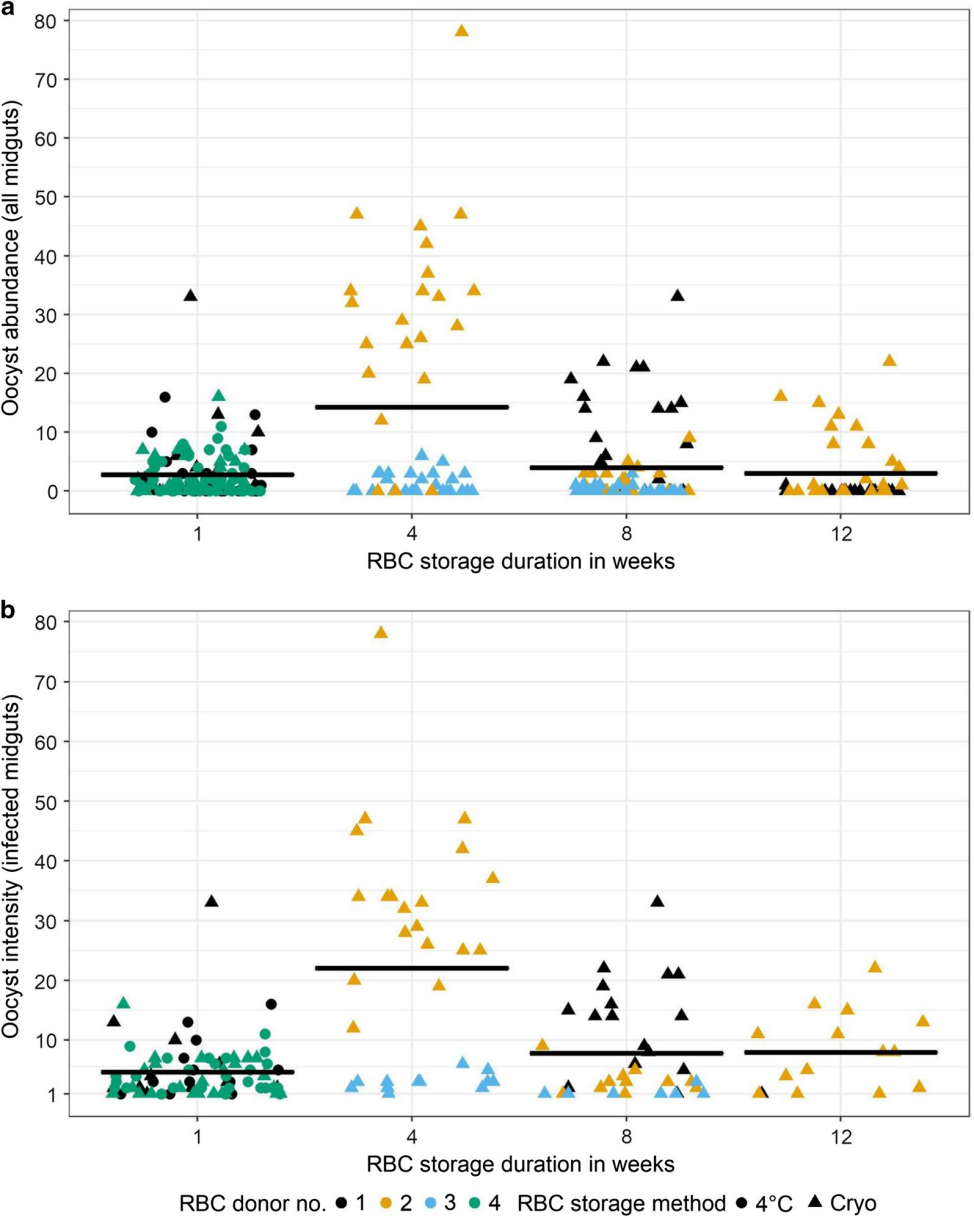
**a** Oocyst abundance (oocyst counts from all midguts regardless of infection status) and **b** intensity (oocyst counts from infected midguts only) in mosquitoes depicted in Fig. 2 (left, top and bottom panels). Parasites were cultured in RBCs collected from 4 independent donors (indicated by colours) stored for 1 week as refrigerated (“4 °C”, circles) or cryo-preserved (“Cryo”, triangles). Horizontal bars represent group means with each data point representing oocyst counts from an individual mosquito midgut. For visualization purposes, counts were jittered horizontally to 40% but not vertically to maintain alignment with the gradient on the y-axis

Oocyst abundance and intensity were used to determine if parasite burdens in the midguts of mosquitoes differed due to storage method or duration. Mean (± se) oocyst abundance (oocyst counts from all sampled midgut) was 5.4 ± 2.53 (n = 11 SMFAs, mean mosquito midguts sampled/SMFA = 26 (range = 17–44), total number of mosquito midguts sampled = 283) while oocyst intensity (oocyst counts from infected midguts only) was 7.75 ± 2.85 (n = 11 SMFAs, range of infected midguts/SMFA = 1–28, total number of mosquito midguts sampled = 157) (Fig. 3). Overall, oocyst abundance (mean ± se) and intensities were 2.73 ± 0.434 (n = 2) and 4.5 ± 0.559 (n = 2) respectively in mosquitoes infected with parasites cultured in refrigerated RBCs and 5.72 ± 0.754 (n = 2) and 10.6 ± 1.23 (n = 9) respectively for parasites in cryo-preserved RBCs.

While both abundance and intensity were independent of storage method (Incidence rates ratio for abundance (IRR) = 1.20, se = 0.71, p = 0. 80, IRR for intensity = 1.12, se = 0.36, p = 0.75) or duration (IRR for abundance = 0.86, se = 0.1, p = 0.13, IRR for intensity = 0.95, se = 0.07, p = 0.48) (Table 1), most of the random variation was generated by the differences between experimental blocks with some contribution from RBC donor-specific characteristics and the least from the variation among infections (Table 1).

### Gametogenesis of Cambodian isolate *P. falciparum* (CB132)

To determine if cryo-preservation can, in principle, support infectious cultures of other strains of *P. falciparum,* RBC donor number four used for the NF54 cultures above (Additional file 2) was used as a substrate for sexual stage cultures of a clinical isolate of Cambodian origin (*P. falciparum* CB132 [30]) following storage periods of six (n = 35 midguts and 23 salivary glands) and eight weeks (n = 49 midguts) (Additional file 6). Statistical analyses were not performed due to the lack of replication in the study design.

## Discussion

In the current study, data from nine SMFAs unequivocally demonstrate that cryo-preserved RBCs provide a suitable substrate for gametocytogenesis of *P. falciparum* in vitro but crucially, the resulting sexually mature gametocytes underwent gametogenesis and sporogony in *An. stephensi* mosquitoes. Additionally, evidence is shown to suggest that *in general*, cryo-preservation did not alter the substrate properties of the RBCs following storage for up to 12 weeks, however, their ability to support infectious gametocytes suggested a gradual but statistically in-significant decline after 12 weeks of storage. Until this trend is confirmed with more RBC donors, it is recommended that the use of cryo-preserved RBCs should be restricted to 8 weeks for SMFAs, especially considering the time and effort required (Fig. 2b).

Although cryogenically preserved RBCs have been shown to be suitable for culturing other *Plasmodium* species, including the asexual stages of *P. falciparum*, none of the studies ran *P. falciparum* cultures for more than 4 days [28, 29]. The results from the current study demonstrate that in addition to supporting proliferation of the asexual stages for the first 4 days, cryo-preserved RBCs were able to maintain their “qualities” as a substrate for the additional 10–12 days required for progression through the various stages of gametocytogenesis. Indeed, while not the focus of this study, it is worth adding that for general maintenance as well as for initiating gametocytogenesis, asexual seed cultures were propagated solely in freshly thawed cryo-preserved RBCs because of reproducible and predictable growth rates (unpublished observations). Neither cryo-preservation nor duration of storage up to 12 weeks affected the ability of cryo-preserved RBCs to provide a substrate for parasite growth and differentiation of sexual stages of *P. falciparum* NF54 into mature gametocytes in vitro. Taken together, the current study suggests that cryo-preservation has the potential to completely replace refrigeration for in vitro cultures of *P. falciparum*, with the added benefit of minimizing RBC donor effects [14, 47].

Rates of gametocytaemia were apparently higher in cryo-preserved RBCs with a decline in concentrations following a peak at 12 days post-seeding (Fig. 1b), however, this non-linearity was accounted for by the random variation between experimental blocks (Table 1). The most obvious difference between the blocks was the growth rates of asexual stages in the asexual feeder flasks used to initiate the gametocyte cultures in each experimental block (Additional file 5a, compare rates in experimental blocks 1 with blocks 2–5). Although statistical models suggested a positive association with the proportions of RBCs infected with parasites at the late trophozoite stage and subsequent rates of gametocytaemia, the current study design and overall objectives precluded more robust analyses of this relationship. Indeed, future experiments with synchronized asexual feeder cultures seeded at various starting densities should help tease apart the underlying relationships, manipulation of which could help design more streamlined regimes for inducing gametocytogenesis. Nevertheless, the relationship between late trophozoites in asexual feeder flasks and future gametocytaemia has been suggested by others [13] and taken together, observations from the current study lend further support to the importance of considering parasite fitness at the asexual stages and subsequent gametocytogenesis in vitro.

The rates of gametocytaemia in vitro were not entirely reflected in the patterns of oocyst prevalence of *P. falciparum* NF54 in midguts of *An. stephensi* mosquitoes. While the ability of cryo-preserved RBCs to support infectious gametocytes suggested a statistically in-significant decline after 12 weeks of storage, the trend was driven by one RBC donor. Whether the observed low prevalence is a donor-specific characteristic would have to be confirmed with more RBC donors representing 12 weeks of cryo-preservation but as stated previously, to be conservative, cryo-preservation periods of ≤ 8 weeks is recommended if the objective is to conduct SMFAs. That being said, refitting statistical models after excluding this infection resulted in a significant reduction in random RBC donor-introduced variation (0.17 in Table 1 to 0.04, remaining output not shown) but less so for the experimental block (0.35 to 0.19) suggesting that in addition to increasing the number of RBC donors, a blocked experimental design similar to the one described here should allow more robust comparisons to determine if RBC donor characteristics are indeed a significant source of variation in oocyst prevalence. Sporozoite prevalence in the salivary glands was independent of storage method and duration and largely mirrors the profiles of oocyst prevalence in the midguts, with the variability in this response variable shaped from inherent variation across the 10 infections. This variability was driven primarily by one infection where prevalence was low following 8 weeks of cryo-preservation. Similarly, neither oocyst abundance nor intensity were affected by maturation of gametocytes in 1-week old refrigerated or one to 12-week old cryo-preserved RBCs. It is important to note that although the terminology used in the current study follows parasitological convention in differentiating between oocyst abundance and intensity [48], “intensity” has also been used to describe abundance in other studies [7, 9].

In general, while cryo-preserved RBCs are clearly suitable for SMFAs, the variability inherent to *P. falciparum* SMFAs [9] implies that the addition of more RBC donors/replication representing duration of storage would not only impart greater confidence in identifying a suitable time-period, but also characterise potential differences between RBC donors not captured by the current study. That being said, preliminary experiments suggest that cryo-preserved RBCs should, in principle, support differentiation of other strains of *P. falciparum*. Six and eight-week old cryo-preserved RBCs from one of the donors previously verified to support gametocytogenesis of NF54 was used to generate transmission-competent gametocytes of a Cambodian isolate of *P. falciparum*, CB132 (Additional file 6) [30]. While future experiments with greater number of donors, storage periods or refrigerated RBCs would be needed to determine and/or optimize cryo-preservation for supporting other strains of *P. falciparum*, collectively, cryo-preservation has the potential to offer benefits similar to the standard NF54 strain, possibly over and above those offered by the current practice of refrigeration.

## Conclusions

In addition to clearly demonstrating the general suitability of using cryo-preserved RBCs for SMFAs with *P. falciparum* at levels similar to the “gold-standard” of using refrigerated 1-week old RBCs, the same RBC population can be cryogenically preserved for up to 8 weeks without affecting their ability to generate transmission-competent gametocytes. The use of cryo-preserved RBCs for SMFAs is not restricted to that of the canonical NF54 isolate of *P. falciparum* and should also be applicable to other strains of *P. falciparum*. From an empirical perspective, the ability to culture parasites in the same donor of RBCs will increase the reproducibility of SMFAs, especially in scenarios where the contribution of RBC donor may warrant consideration. From an economic perspective, 8 weeks offers a significant improvement over the current practice of using RBCs that are ≤ 1-week old, with the potential to reduce the costs by almost sevenfold. Considering how this period also aligns with the 2-month duration suggested for storing blood for maintaining breeder colonies of mosquitoes ([38] and unpublished observations), it is conceivable for laboratories to integrate SMFAs using cryo-preserved RBCs into the general mosquito rearing schedule.

In a broader context, if growth and differentiation of *P. falciparum* is reduced in RBCs that have been refrigerated for different durations, i.e., > 1-week, simultaneous comparisons with cryo-preserved blood may help identify physico-chemical changes in blood that are “naturally” detrimental to parasite fitness. In fact, the adverse effects of prolonged storage of refrigerated RBCs in some cases has led the FDA to recommend identifying the true shelf-life of refrigerated RBCs and towards this end, clinical researchers have started using “omics” technologies to extensively catalog storage-related physico-chemical artefacts, some of which could indeed serve as suitable starting points for comparison [23, 25]. Along the same lines, cryo-preservation has the potential to offer a superior alternative to using older, refrigerated RBCs where “storage-related artefacts”, possibly compounded by inherent differences in RBCs between donors, may influence the interpretation of assays quantifying parasite fitness and/or reproducibility across labs (e.g. comparing mutant strains or testing for drug susceptibility) [14]. Finally, if differences between RBC donors are reflected in the mechanisms and consequences of pathogenesis for instance, cryo-preservation would provide an ideal tool for replicating the observations or allow testing other parasite strains [47].

In summary, the approach described here should be compatible with a broad range of research questions involving the biology of gametocytogenesis as well as human-to-mosquito transmission, with significant potential for integrating into existing research pipelines in addition to identifying novel avenues for target identification and therapeutic intervention. Blocking transmission of the parasite to the mosquito is predicted to be one of the last remaining hurdles to overcome before declaring a malaria-free world [1, 2]. Crucial to this endeavour will be the utilization of SMFAs, the definitive measure of parasite fitness, in an economical and reproducible manner.

## Additional files

**Additional file 1.** A schematic of the overall study design. Description of data: RBCs 3-4 days old post-collection were either refrigerated at 4 °C or cryo-preserved in the gaseous phase of liquid nitrogen. Aliquots of RBCs were thawed at 1, 4, 8 and 12 weeks and assessed for their ability to support 1) gametocytogenesis of *P. falciparum* NF54 *in vitro* and 2) gametogenesis *in vivo* relative to refrigerated RBCs which served as the reference (dashed arrows). Black continuous arrows indicate procedures that were common to all treatments.

**Additional file 2.** Schematic depicting the “partially nested” experimental design wherein not all donors were represented equally across all treatments, with donor-specific treatments identified by the colour-matched text-boxes. Description of data: Schematic depicting the 5 experimental blocks for comparing 1) storage method (“4 °C”, filled circles) and cryo-preservation (“Cryo”, filled triangles) and 2) duration (1-, 4-, 8- and 12-weeks) with coloured symbols indicating RBC donor(s). Each experimental block was defined by the following steps: 1) the *same asexual seed culture* was used to initiate gametocytogenesis, 2) rates of gametocytaemia monitored over 14-16 days in individual flasks with refrigerated (4 °C) and/or cryo-preserved RBCs (i.e., 1 flask/treatment), and 3) sexually mature gametocytes from each flask/treatment offered to *mosquitoes from the same cohort* to assess competence for transmission (see “Study design” under Materials and Methods). However, logistical constraints meant the experimental design turned out be “partially nested” wherein not all 4 donors were represented equally across all treatments. For instance, RBCs from donors “1” and “4” formed the basis for simultaneous comparison of storage method in blocks 1 and 5 wherein the same starting asexual seed culture was used to initiate gametocytogenesis in separate flasks (1 flask for each storage method) with the resulting mature gametocytes used in SMFAs with separate groups of mosquitoes but originating from the same cohort. Additionally, donor “1” was re-tested following cryogenic storage for 8 and 12-weeks (but not 4-weeks) within experimental blocks 2 and 3 respectively, along with donor “2”, with the exception that RBCs from the latter donor had been cryogenically preserved for 4 and 8 weeks, respectively.

**Additional file 3.** Representative images used to classify the various stages of *P. falciparum* NF54 during this study. Description of data: a.) Giemsa-stained images of male gametocytes (1000x, oil immersion, brightfield), b.) Giemsa-stained images of female gametocytes (1000x, oil immersion, brightfield), c.) Ex-flagellation of gametocytes *in vitro* with arrowheads depicting flagella (400x DIC, unstained), d.) An oocyst with enclosed sporozoites (arrowheads, 400x, DIC, unstained), e.) ruptured salivary glands with freed sporozoites (arrowheads) at 100x (i.) and 400x (ii) (DIC, unstained). Images were captured with an LG G3 or Samsung Galaxy S7 smartphone using default settings (“Auto”) while attached to the eyepieces on the microscope with a custom-designed apparatus. Except panel e, all images were digitally magnified up to 4x for presentation purposes.

**Additional file 4.** Rates of development for the various immature stages of gametocytes of *P. falciparum* NF54 cultured *in vitro* in refrigerated (solid line) or cryo-preserved (dashed line) RBCs with the top panel showing stage II gametocytaemia (a), stage III (b) and lastly, stage IV gametocytaemia preceding the appearance of sexually mature gametocytes. Data represents mean ± standard errors (se).

**Additional file 5.** Variation between experimental blocks and RBC donor in (a) the rates of mature gametocytaemia *in vitro* and b) infectiousness of resulting gametocytes as estimated by prevalence of *P. falciparum* NF54 oocysts in midguts of *An. stephensi.* Description of data: a) rates of *in vitro* gametocytaemia within an experimental block depicting RBC donor-specific trends (colours) for comparing storage treatment (“4 °C”, filled circles and cryo-preservation (“Cryo”, filled triangles) or duration (1-, 4-, 8- or 12-weeks indicated by text box in the same colour scheme as the respective donor. Each line connecting data points represent gametocytaemia from a single flask monitored at the indicated days post-seeding. (b.) The infectiousness of resulting sexually mature gametocytes as measured by corresponding SMFA within the same block with shapes and colours indicating the same parameters but with labels excluding “weeks” due to space constraints. Horizontal bars represent mean prevalence within each block.

**Additional file 6.** Cryo-preserved RBCs support SMFAs with a Cambodian isolate of *P. falciparum*. Description of data: a) Oocyst and sporozoite prevalence, b) oocyst abundance and c) intensity of *P. falciparum* CB132 in female *An. stephensi* infected with mature gametocytes of *P. falciparum* CB132 cultured in RBCs from donor “4” (Additional file 2) thawed following cryo-preservation for 6 (left panel) or 8 weeks (right panel). Horizontal bars represent group means with each data point representing oocyst counts from an individual mosquito midgut. For visualization purposes, counts were jittered horizontally to 40% but not vertically to maintain alignment with the gradient on the y-axis. NA=not available.

## Abbreviations

DIC: differential interference contrast
GLMMs: generalized linear mixed-effects models
IRR: incidence rates ratios
OR: odds ratios
SMFAs: standard membrane feeding assays
