# Supplementary figures and images for: Cryogenically preserved RBCs support gametocytogenesis of Plasmodium falciparum in vitro and gametogenesis in mosquitoes
Source: Malar J. 2018 Dec 6;17:457. doi: 10.1186/s12936-018-2612-y (PMC6282341; doi:10.1186/s12936-018-2612-y)

# Additional file 1

## Fresh RBCs, 3-4 days/<1 week post-collection

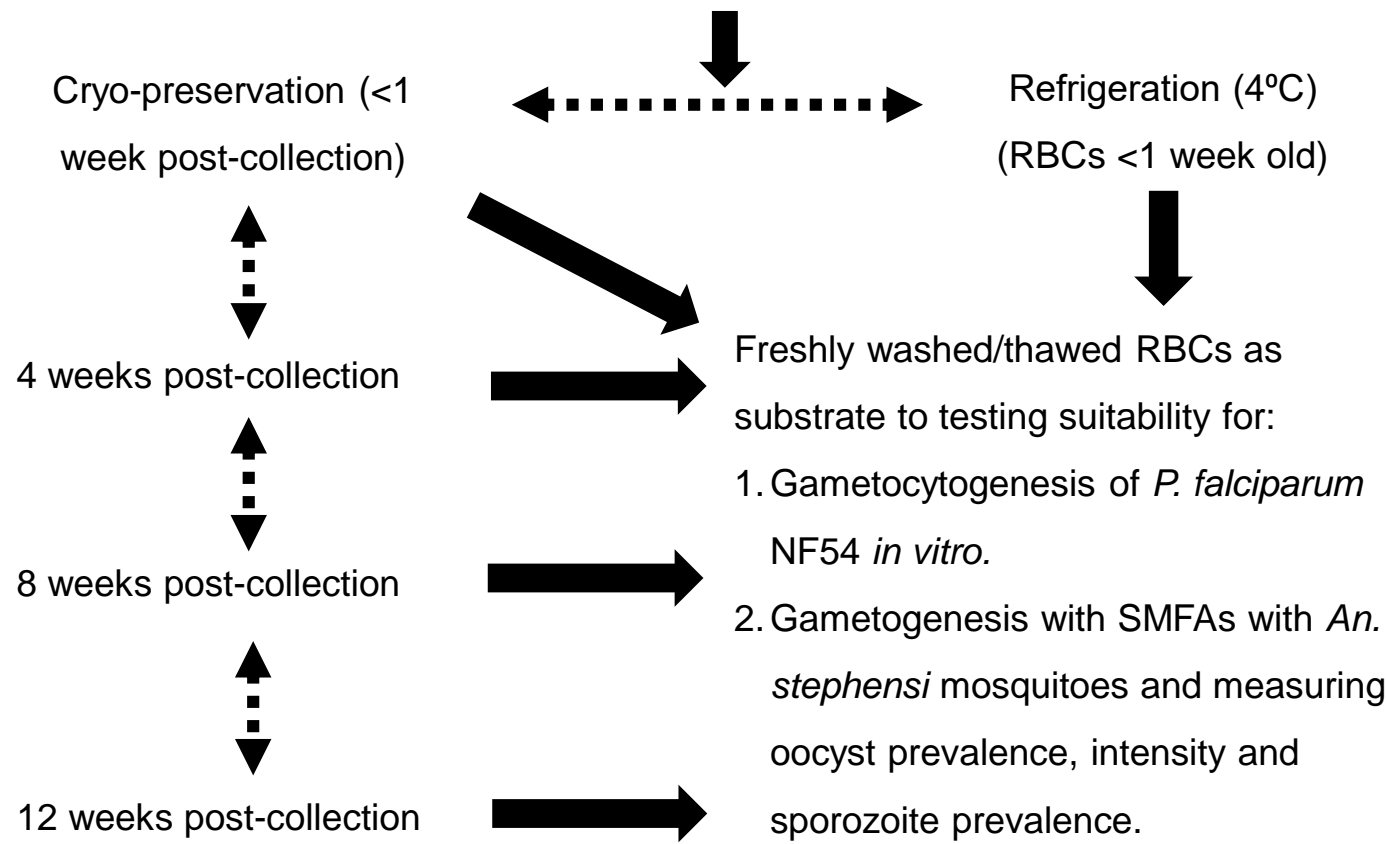

Supplement: Supplementary file 1 — Additional file 1. A schematic of the overall study design. Description of data: RBCs 3-4 days old post-collection were either refrigerated at 4 °C or cryo-preserved in the gaseous phase of liquid nitrogen. Aliquots of RBCs were thawed at 1, 4, 8 and 12 weeks and assessed for their ability to support 1) gametocytogenesis of P. falciparum NF54 in vitro and 2) gametogenesis in vivo relative to refrigerated RBCs which served as the reference (dashed arrows). Black continuous arrows indicate procedures that were common to all treatments. [file 12936_2018_2612_MOESM1_ESM.pdf]

Additional file 2

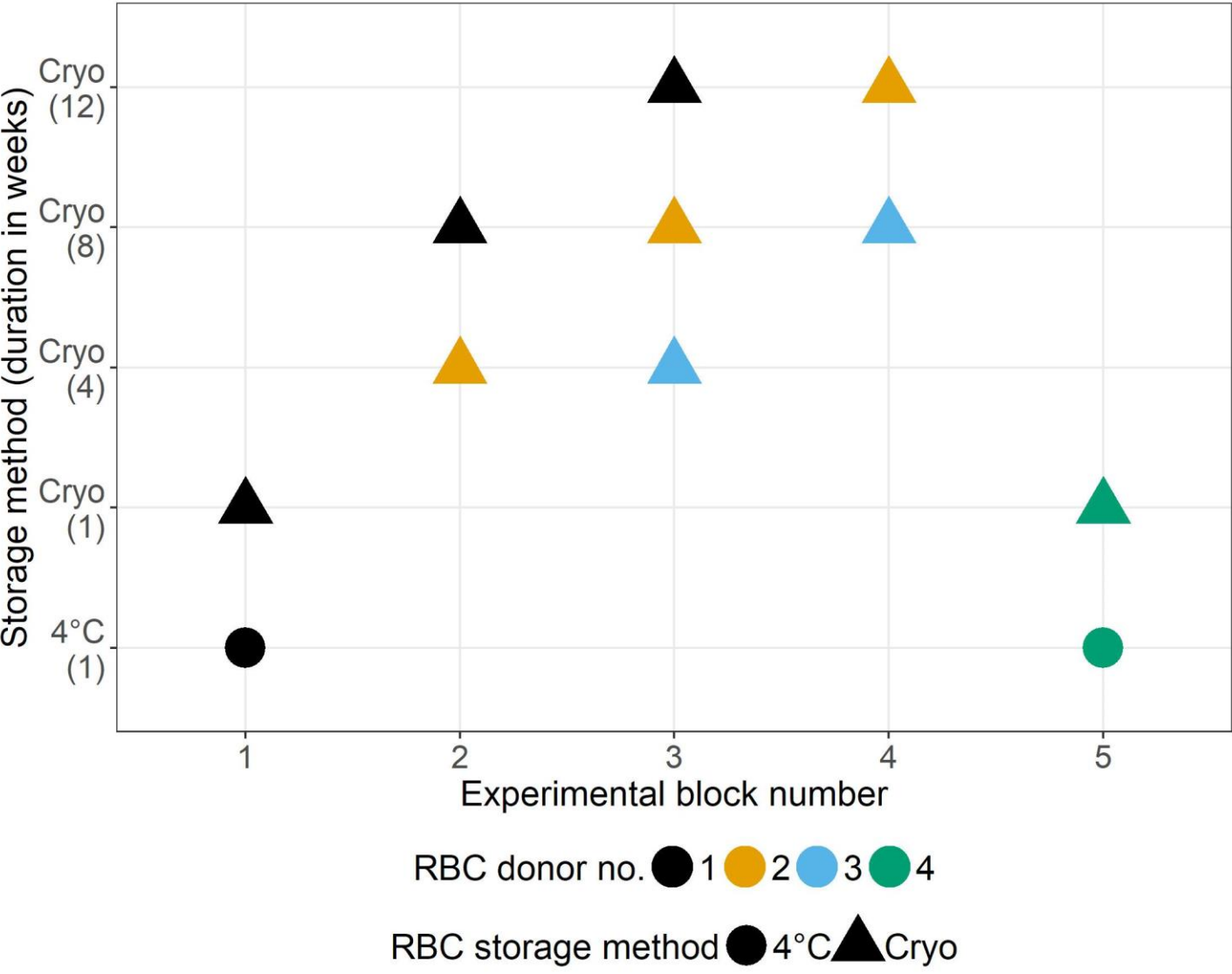

Supplement: Supplementary file 2 — Additional file 2. Schematic depicting the “partially nested” experimental design wherein not all donors were represented equally across all treatments, with donor-specific treatments identified by the colour-matched text-boxes. Description of data: Schematic depicting the 5 experimental blocks for comparing 1) storage method (“4 °C”, filled circles) and cryo-preservation (“Cryo”, filled triangles) and 2) duration (1-, 4-, 8- and 12-weeks) with coloured symbols indicating RBC donor(s). Each experimental block was defined by the following steps: 1) the same asexual seed culture was used to initiate gametocytogenesis, 2) rates of gametocytaemia monitored over 14-16 days in individual flasks with refrigerated (4 °C) and/or cryo-preserved RBCs (i.e., 1 flask/treatment), and 3) sexually mature gametocytes from each flask/treatment offered to mosquitoes from the same cohort to assess competence for transmission (see “Study design” under Materials and Methods). However, logistical constraints meant the experimental design turned out be “partially nested” wherein not all 4 donors were represented equally across all treatments. For instance, RBCs from donors “1” and “4” formed the basis for simultaneous comparison of storage method in blocks 1 and 5 wherein the same starting asexual seed culture was used to initiate gametocytogenesis in separate flasks (1 flask for each storage method) with the resulting mature gametocytes used in SMFAs with separate groups of mosquitoes but originating from the same cohort. Additionally, donor “1” was re-tested following cryogenic storage for 8 and 12-weeks (but not 4-weeks) within experimental blocks 2 and 3 respectively, along with donor “2”, with the exception that RBCs from the latter donor had been cryogenically preserved for 4 and 8 weeks, respectively. [file 12936_2018_2612_MOESM2_ESM.pdf]

**Additional file 3a**

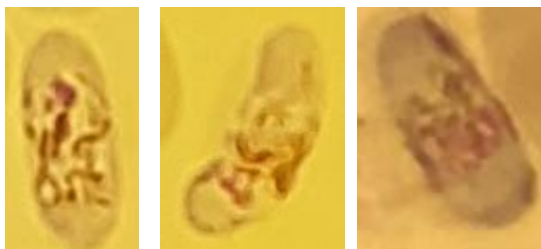

**Additional file 3b**

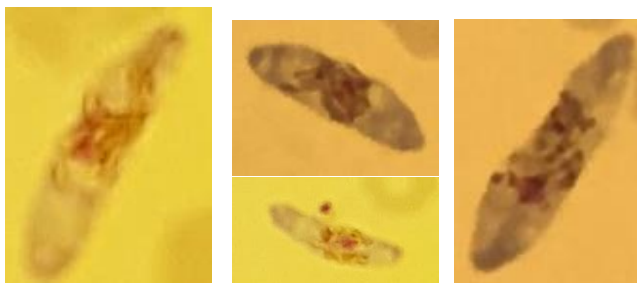

**Additional file 3c**

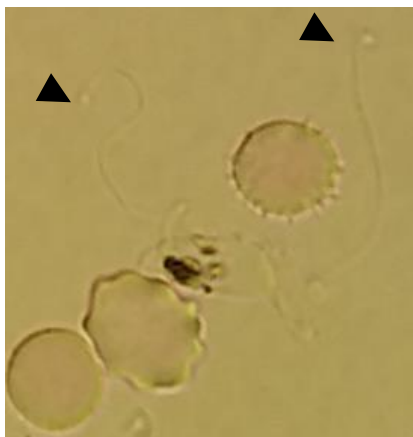

**Additional file 3d**

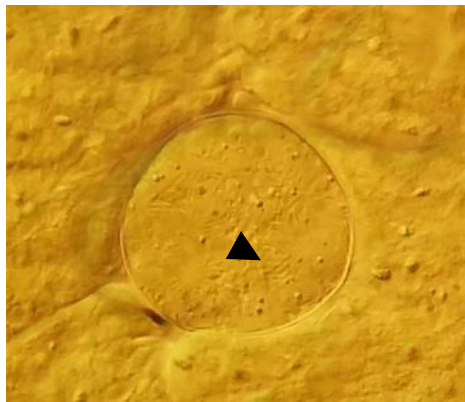

**Additional file 3e**

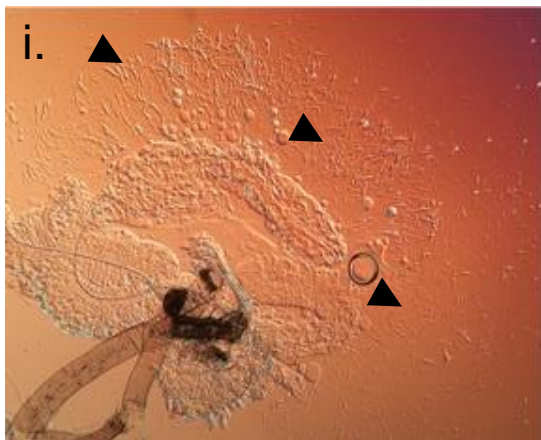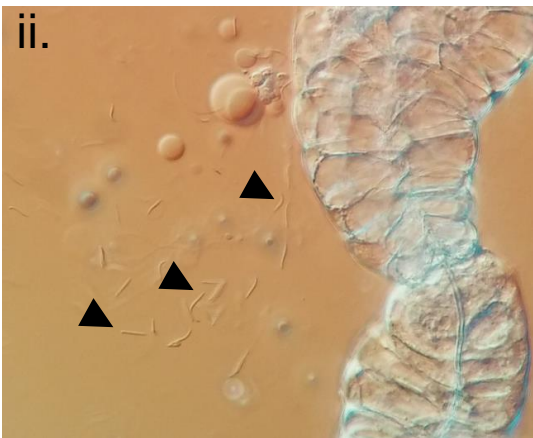

Supplement: Supplementary file 3 — Additional file 3. Representative images used to classify the various stages of P. falciparum NF54 during this study. Description of data: a.) Giemsa-stained images of male gametocytes (1000x, oil immersion, brightfield), b.) Giemsa-stained images of female gametocytes (1000x, oil immersion, brightfield), c.) Ex-flagellation of gametocytes in vitro with arrowheads depicting flagella (400x DIC, unstained), d.) An oocyst with enclosed sporozoites (arrowheads, 400x, DIC, unstained), e.) ruptured salivary glands with freed sporozoites (arrowheads) at 100x (i.) and 400x (ii) (DIC, unstained). Images were captured with an LG G3 or Samsung Galaxy S7 smartphone using default settings (“Auto”) while attached to the eyepieces on the microscope with a custom-designed apparatus. Except panel e, all images were digitally magnified up to 4x for presentation purposes. [file 12936_2018_2612_MOESM3_ESM.pdf]

**Additional file 4a**

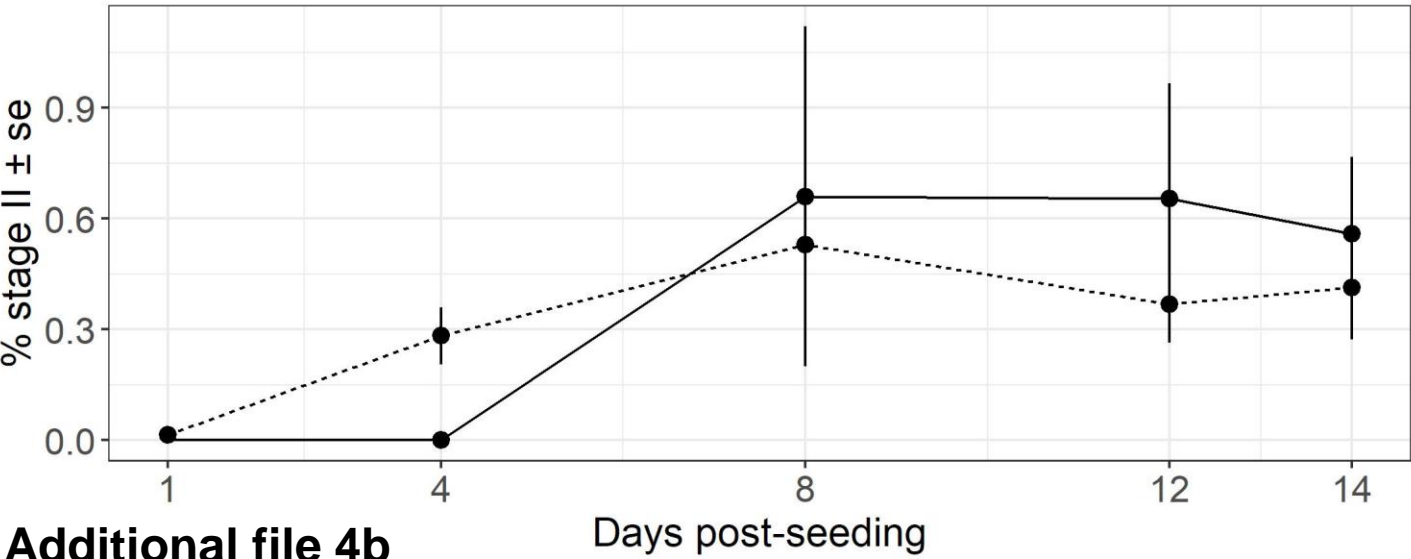

**Additional file 4b**

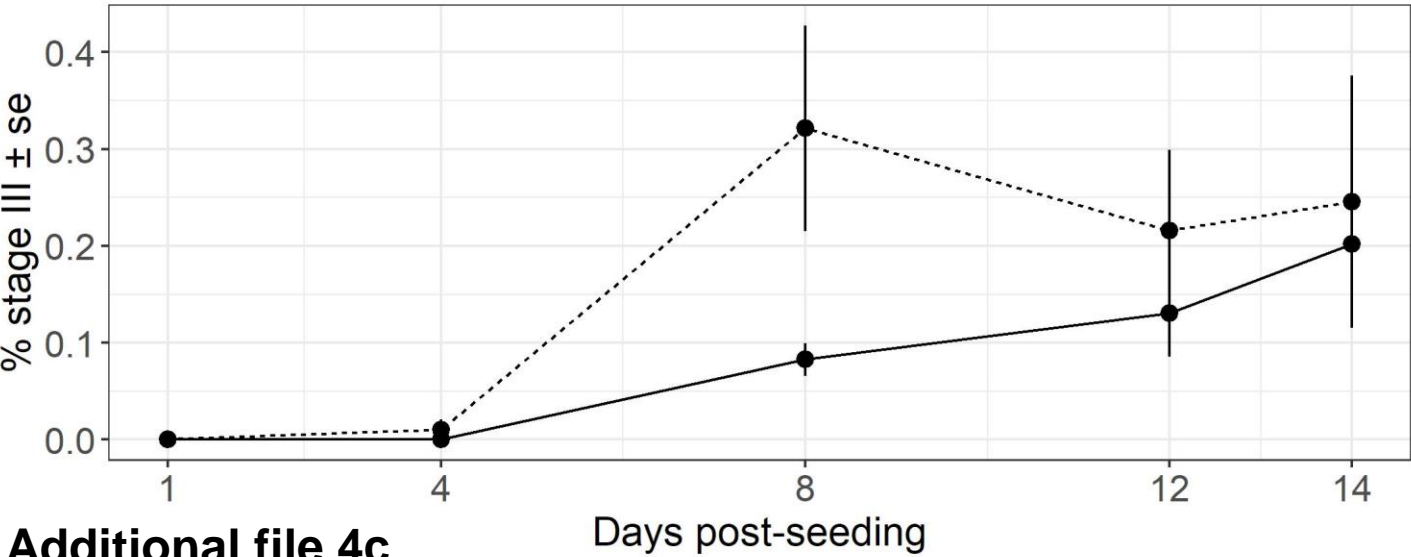

**Additional file 4c**

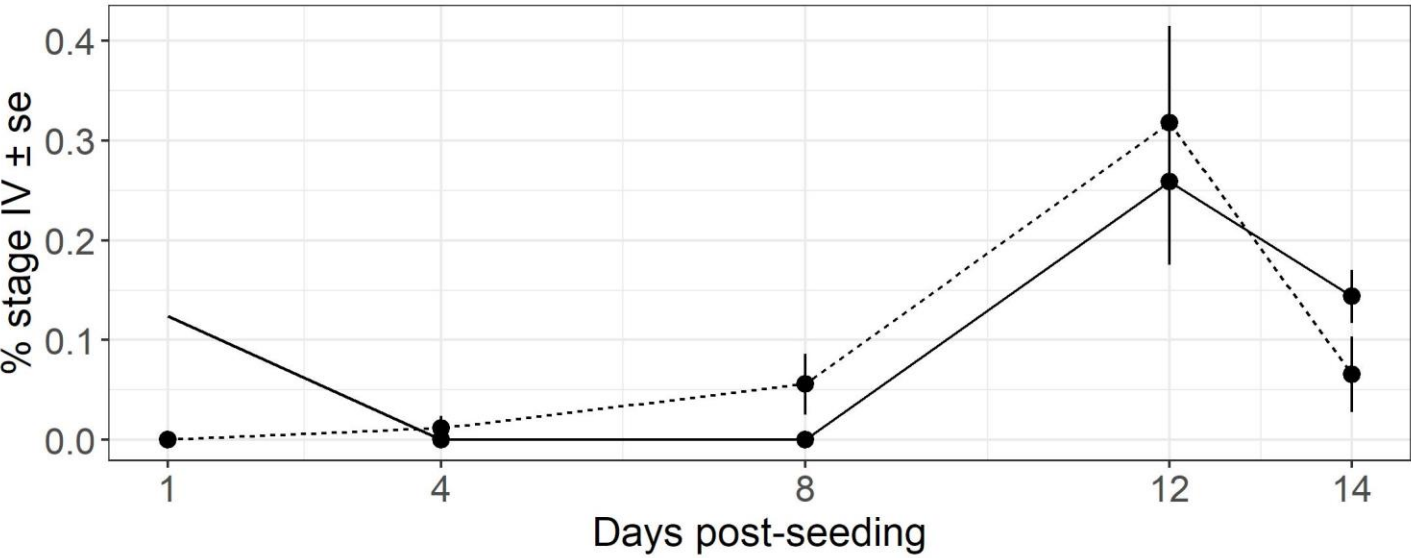

Supplement: Supplementary file 4 — Additional file 4. Rates of development for the various immature stages of gametocytes of P. falciparum NF54 cultured in vitro in refrigerated (solid line) or cryo-preserved (dashed line) RBCs with the top panel showing stage II gametocytaemia (a), stage III (b) and lastly, stage IV gametocytaemia preceding the appearance of sexually mature gametocytes. Data represents mean ± standard errors (se). [file 12936_2018_2612_MOESM4_ESM.pdf]

Additional file 5a

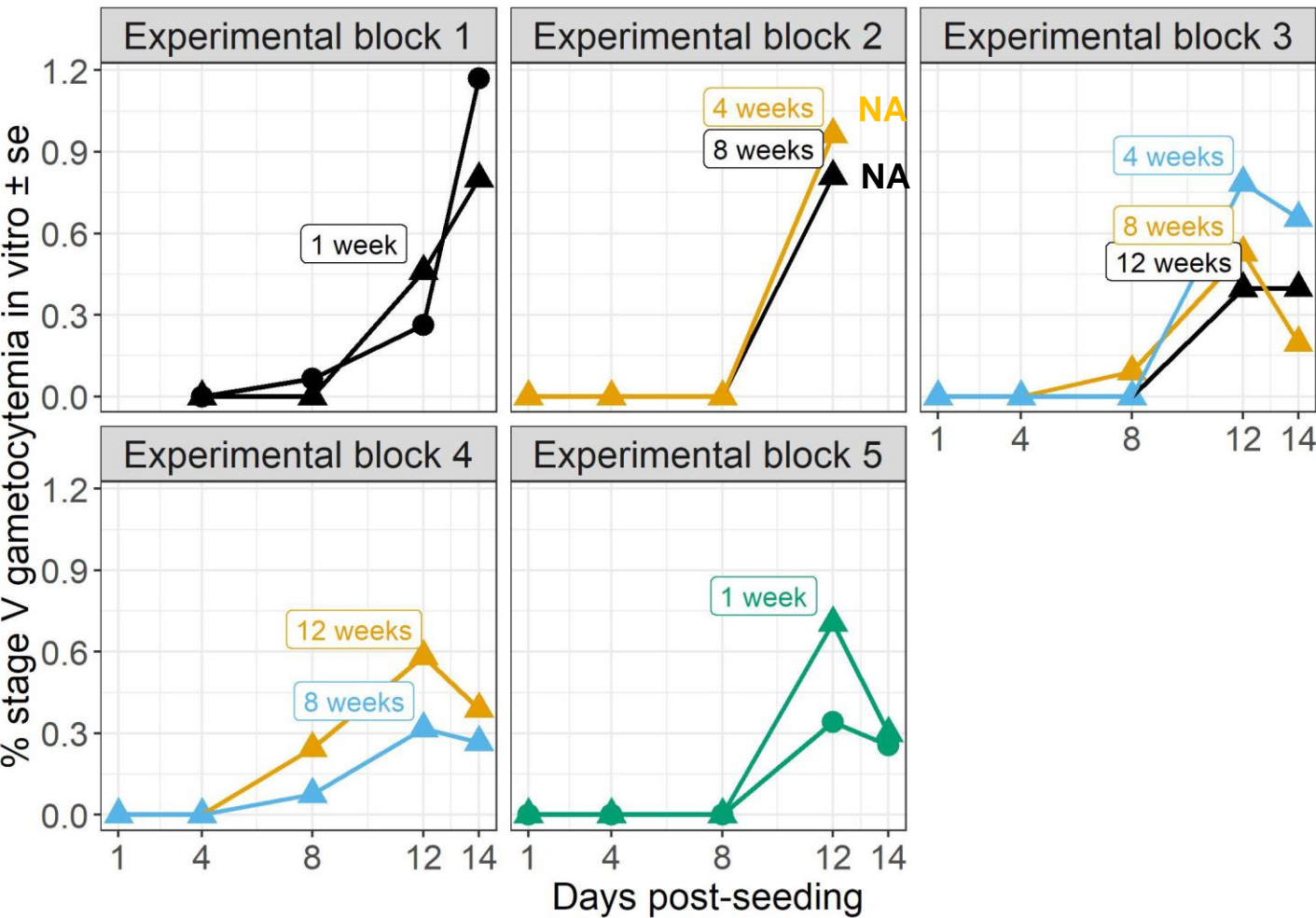

RBC donor no. ● 1 ● 2 ● 3 ● 4 RBC storage method ● 4°C ▲ Cryo

Additional file 5b

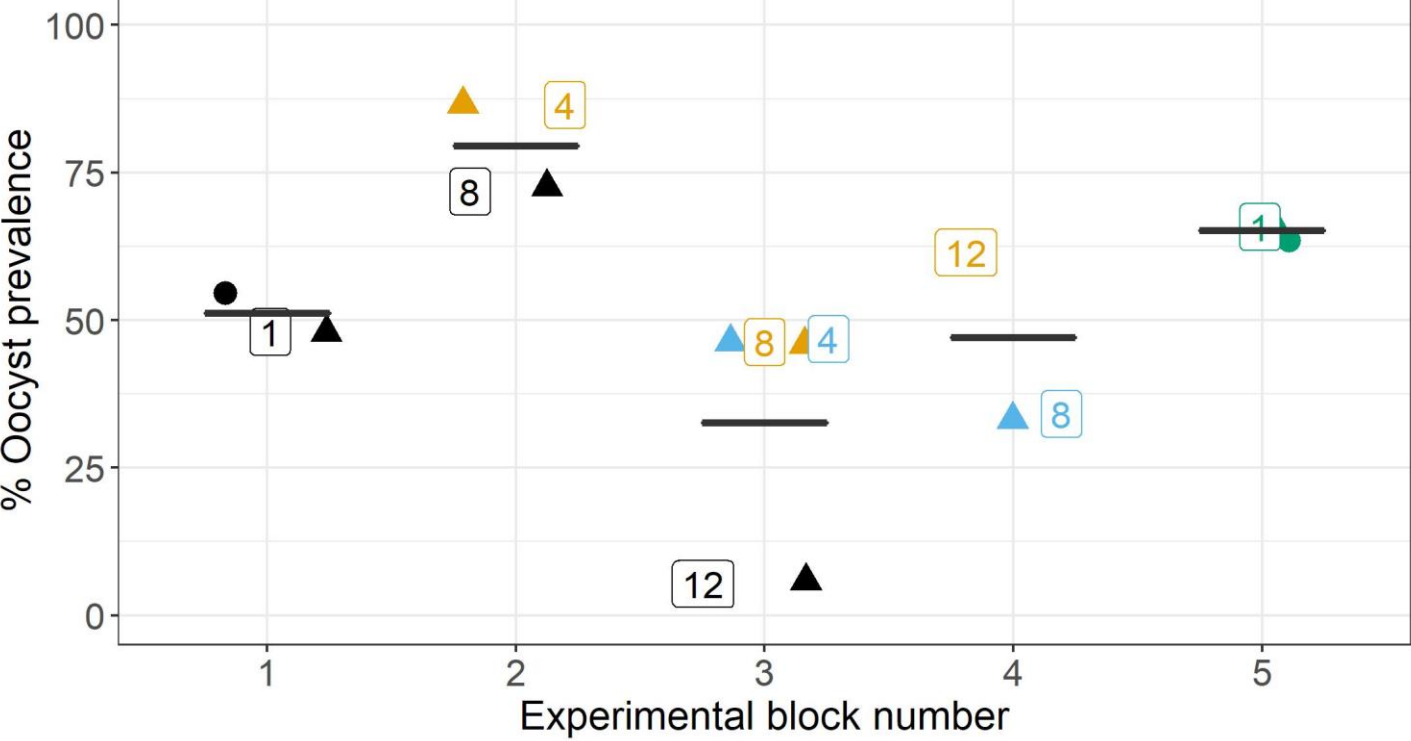

Supplement: Supplementary file 5 — Additional file 5. Variation between experimental blocks and RBC donor in (a) the rates of mature gametocytaemia in vitro and b) infectiousness of resulting gametocytes as estimated by prevalence of P. falciparum NF54 oocysts in midguts of An. stephensi. Description of data: a) rates of in vitro gametocytaemia within an experimental block depicting RBC donor-specific trends (colours) for comparing storage treatment (“4 °C”, filled circles and cryo-preservation (“Cryo”, filled triangles) or duration (1-, 4-, 8- or 12-weeks indicated by text box in the same colour scheme as the respective donor. Each line connecting data points represent gametocytaemia from a single flask monitored at the indicated days post-seeding. (b.) The infectiousness of resulting sexually mature gametocytes as measured by corresponding SMFA within the same block with shapes and colours indicating the same parameters but with labels excluding “weeks” due to space constraints. Horizontal bars represent mean prevalence within each block. [file 12936_2018_2612_MOESM5_ESM.pdf]

## Additional file 6a

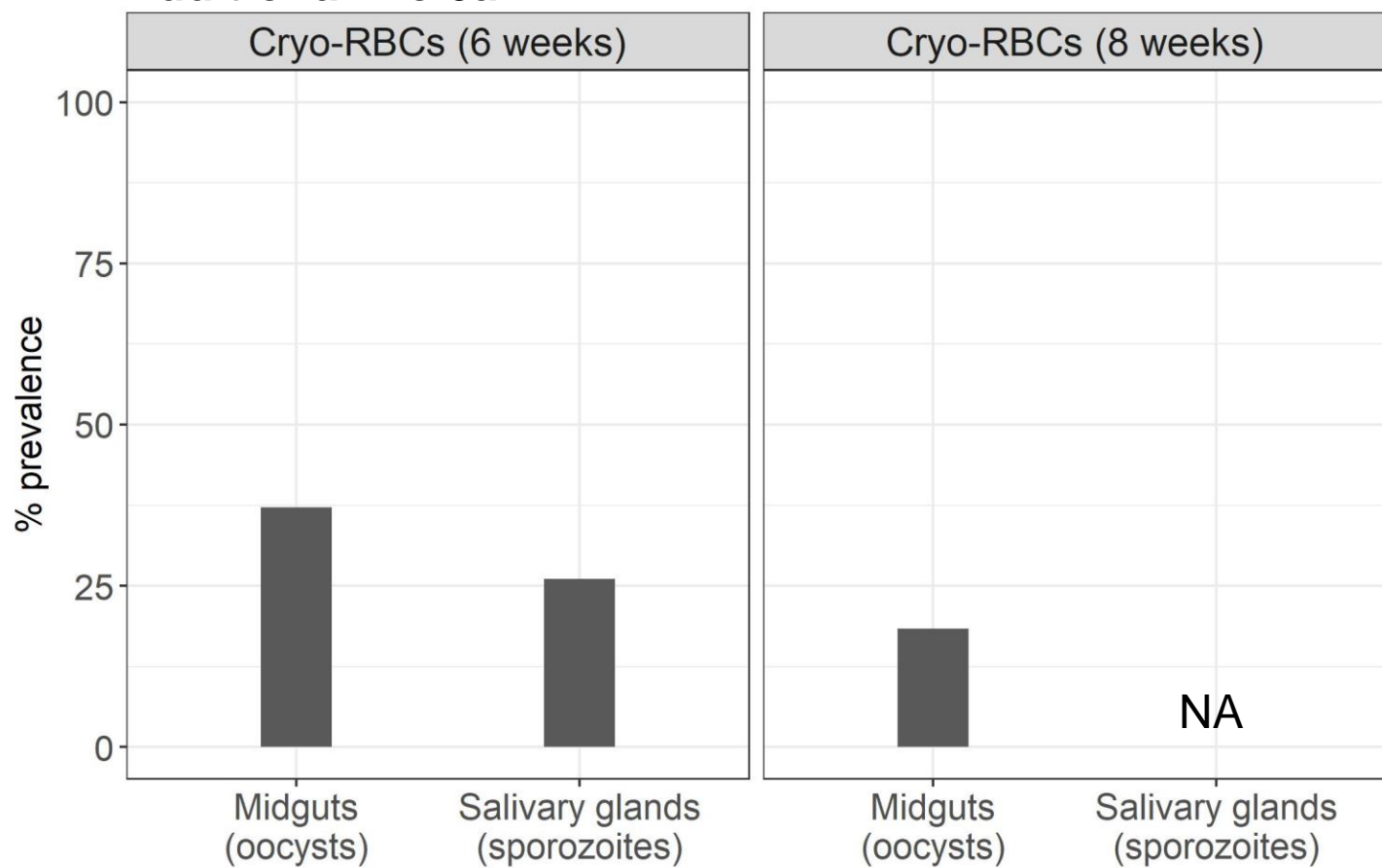

## Additional file 6b

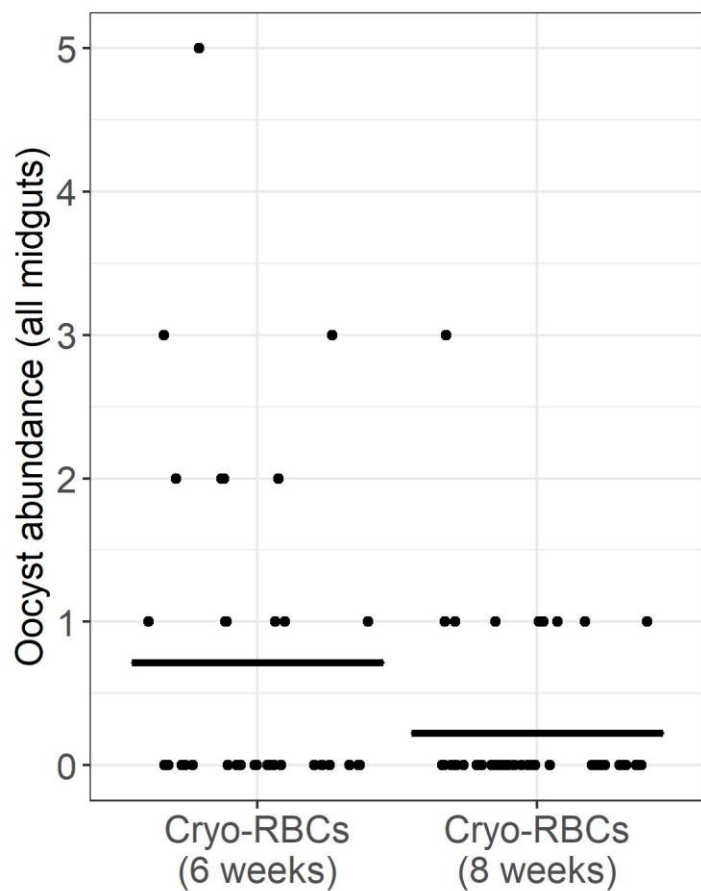

### Additional file 6c

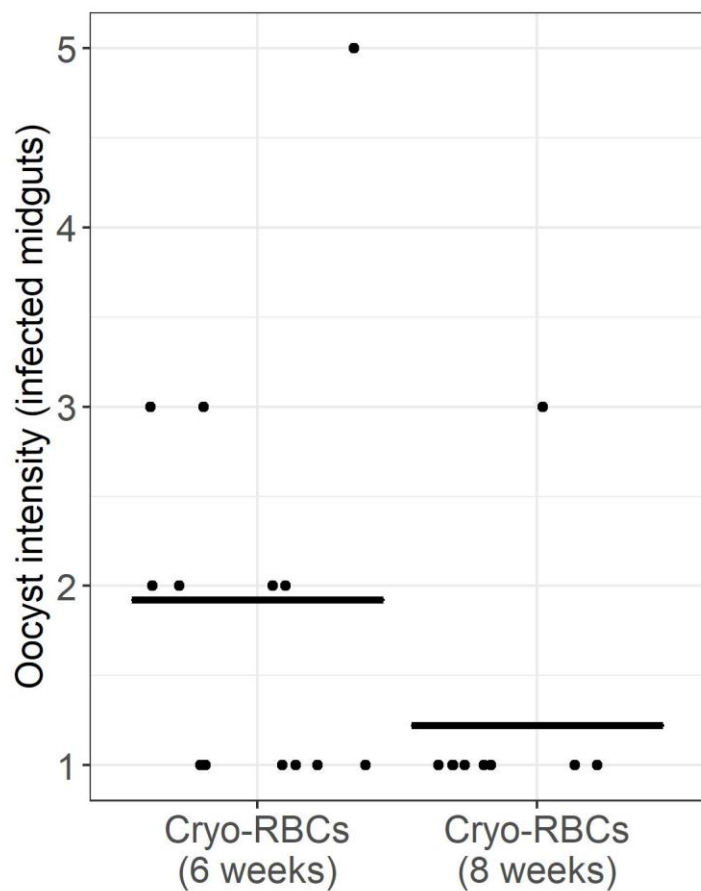

Supplement: Supplementary file 6 — Additional file 6. Cryo-preserved RBCs support SMFAs with a Cambodian isolate of P. falciparum. Description of data: a) Oocyst and sporozoite prevalence, b) oocyst abundance and c) intensity of P. falciparum CB132 in female An. stephensi infected with mature gametocytes of P. falciparum CB132 cultured in RBCs from donor “4” (Additional file 2) thawed following cryo-preservation for 6 (left panel) or 8 weeks (right panel). Horizontal bars represent group means with each data point representing oocyst counts from an individual mosquito midgut. For visualization purposes, counts were jittered horizontally to 40% but not vertically to maintain alignment with the gradient on the y-axis. NA=not available. [file 12936_2018_2612_MOESM6_ESM.pdf]
